# Predicting the consequences of physical activity: An investigation into the relationship between anxiety sensitivity, interoceptive accuracy and action

**DOI:** 10.1371/journal.pone.0210853

**Published:** 2019-03-28

**Authors:** Abby Tabor, Niels Vollaard, Edmund Keogh, Christopher Eccleston

**Affiliations:** 1 Centre for Pain Research, Department for Health, University of Bath, Bath, United Kingdom; 2 Department for Health, University of Bath, Bath, United Kingdom; 3 Department of Clinical and Health Psychology, Ghent University, Ghent, Belgium; Anglia Ruskin University, UNITED KINGDOM

## Abstract

The ability to predict the consequences of our actions is imperative for the everyday success of our interactions. From negotiating an uneven surface, to mounting an immune response, we continually infer the limits of our body. The current investigation considered the impact that the inferred consequences of action has on the placement of limits. We hypothesised that the performance of individuals in a novel, sprint task would reflect both their ability to accurately detect changes in bodily arousal (Interoceptive Accuracy) and the inferred consequences associated with heightened arousal signals (Anxiety Sensitivity). We found that individuals who demonstrated accuracy associated with physiological arousal changes, *and* who showed a heightened fear of the consequences of arousal symptoms, modified their actions by decreasing their power output (mean Watts•kg^-1^) in a sprint task (Δ*R*^*2*^ = 0.19; *F*(1,34) = 19.87); *p*<0.001). These findings provide a basis for understanding the varying actions taken as we encounter bodily perturbation.

## Introduction

As we negotiate our environment, we are continually required to disambiguate noisy and uncertain information [1]. The actions of our body, and the continual predictions of the consequences of these actions, help us to make sense of a changing world [2]. We are able to actively filter information and flexibly update our predictions as our body and the environment change [3]. Sensing changes specific to the body is known as interoception, and it is comprised of 3 dimensions: *Interoceptive accuracy*- objective performance in detection; *Interoceptive sensibility-* self-evaluated assessment of interoception; *Interoceptive awareness-* metacognitive appraisal of accuracy [4]. It has been proposed that the ability to precisely detect changes in our body—Interoceptive Accuracy—promotes flexible updating of the predictions of our body and allows for a more fine-tuned regulation of behaviour [5–9].

Despite evidence for the role that Interoceptive Accuracy plays in influencing bodily regulation, the mechanism by which heightened Interoceptive Accuracy influences subsequent defensive behaviour is not clear. This is particularly evident when considering the dichotomous associations that have been drawn from heightened Interoceptive Accuracy; considered on the one hand to be highly advantageous in contexts such as elite sport [10,11] and on the other as a contributing factor in the manifestation of psychopathology [12,13]. This contention may, in part, be attributed to the methods by which we are able to quantify Interoceptive Accuracy. There is an ongoing need to reduce potential confounders, such as attention and expectation, obscuring the true nature of interoceptive accuracy [14].

Despite the inconsistency in the literature, there appears a clear challenge to the idea that the accuracy with which we detect bodily changes directly influences action. Indeed, there is mounting evidence for the role of inference and prediction in the determination of action, specifically the inferred consequences associated with identified changes to the body [15,16].

**Fig 1:**
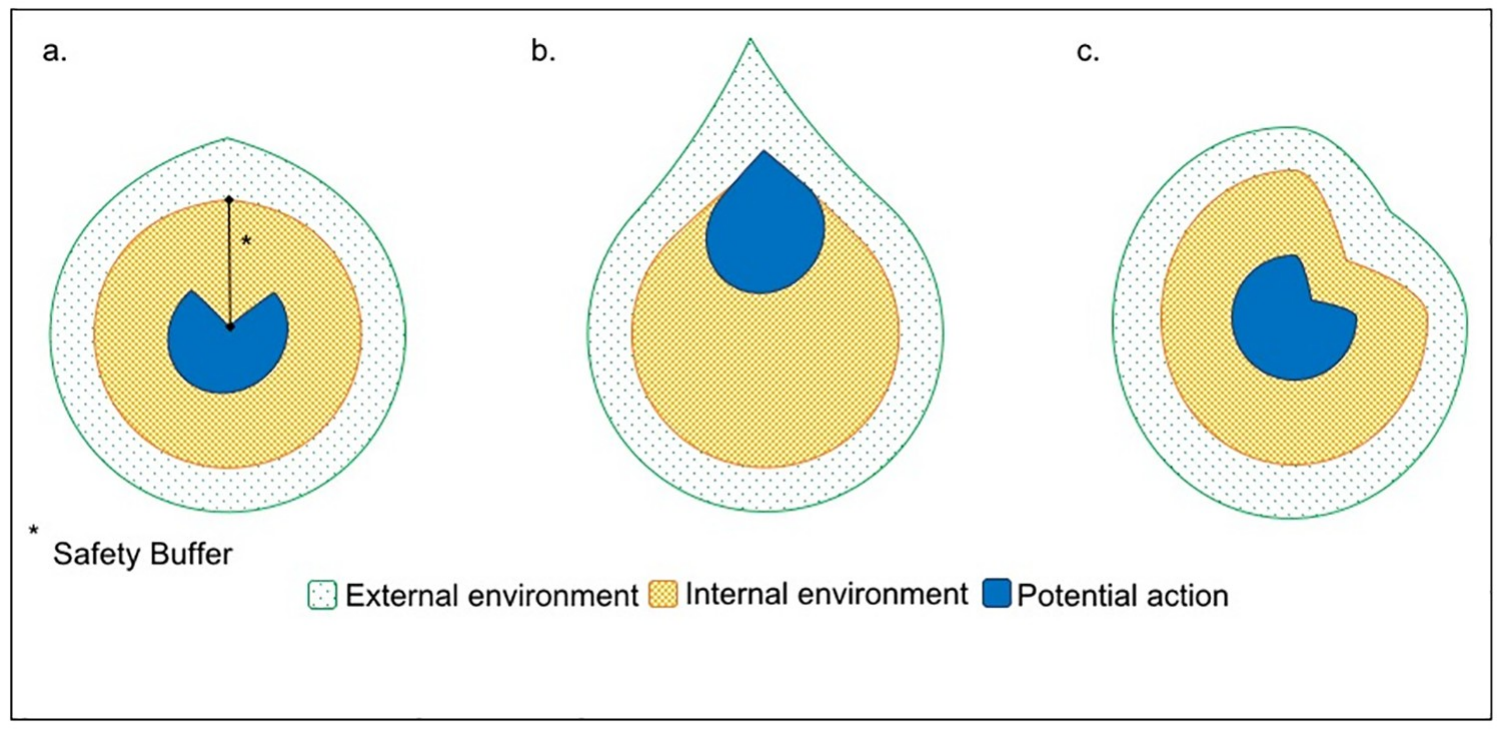
Schematic representation of altered ‘safety-buffer’ and potential action in different individuals. a) An individual with high accuracy related to their internal or body-specific environment, but also with high fear associated with arousal related signals. Placed in a novel environment (green), e.g., a sprint exercise task, they are required to infer the state of their body (orange) in relation to their environment and predict the consequences of potential action within the task. In this case, potential action is reduced (blue) to minimise the change in bodily arousal, a consequence of action that is associated with fear. b) An elite athlete, placed in a familiar environment (green) e.g., a sprint exercise task. As part of their training they are familiar with the changes in bodily arousal (orange) that occur as part of a sprint task and have refined predictions about the consequences of their potential actions (blue). In this case, the athlete is able to push bodily limits, reducing the ‘safety-buffer’ and enhancing the action output. c) An individual with a sprained ankle is placed in a known environment (green). As a consequence of their injury, their bodily state (orange) is altered, and even familiar environments become challenging. They must predict the consequences of their actions, e.g., weight-bearing through the ankle, and infer the appropriate potential actions (blue). In acute injury, these potential actions are reduced to promote recovery.

From an evolutionary perspective, the ability to accurately infer and predict the consequences of bodily change is imperative to the everyday success of negotiating a changing environment [2]. This may be as routine as predicting the depth of a chair as we sit, or as complex as attempting to ascertain the threat posed by a potentially injured back. In both cases, actions are continually informed by our predicted consequences of action. This process can be broadly conceptualised as a trade-off between when to approach and when to defend [17], reflective of a changing bodily ‘safety-buffer’- the margin between absolute bodily limits and inferred bodily limits (Fig 1).

In the case of conditions that involve a prolonged period of *defensive action*, such as anxiety, panic, or pain, it has been observed that individuals are fearful of the predicted consequences of bodily experiences, particularly arousal-based changes of the body (Fig 1c) [18,19], and can engage in action that perpetuates the condition, e.g. avoidance [20–22]. Fear of the predicted consequences of bodily experiences, such as arousal-related changes in heart rate, has been widely studied in relation to a range of conditions, such as panic [19,23–26] and pain (e.g., [8,18]). For some, chest pain, or simply an increase in heart rate, may be interpreted as an aversive, predictive cue of a ‘heart attack’; for others, dismissed as unrelated to danger. Although the underlying stimulus may be similar the action that is engaged may vary considerably, moderated by the inference of safety or threat, approach or defend [27].

Alternatively, in the case of conditions that involve prolonged *approach action*, such as repetitive training in elite sport, individuals push the boundaries of human performance. Rather than fear of the predicted consequences of bodily change, elite sports performers repeatedly engage in action that attempts to diminish the bodily ‘safety-buffer’ (Fig 1b) [28–30].

Although defence and approach actions manifest differently, the mechanism by which one attempts precise prediction in the context of uncertain outcomes is common to both. Individuals adopt a bodily safety-buffer that is dependent on the predictions of bodily consequences; on the one hand promoting *defence*, on the other, *approach*. Over time, both the individual with a panic disorder and the elite athlete refine their predictions such that they become increasingly precise, yet at both extremes precision does not equate to accuracy. The individual with a panic disorder may hold highly precise predictions of threat, evoking a powerful need to act defensively, but they may be inaccurate in their precision. This potential overestimation of the required safety-buffer has the advantage of never missing a false negative, yet carries the cost of disengaging the individual from their environment. Conversely, the athlete may gain increasingly precise predictions about advancing the limits of their body over time, which promotes successful performance, yet underestimating the safety-buffer carries the cost of performance failure and/or injury.

According to this account, the reciprocal relationship between action and prediction continually attempts to accommodate the inherent uncertainty of the body and the world with increasing finesse [31–33]. Initially, when faced with unfamiliar environments we possess crude, unrefined predictions of the consequences of our actions. However, as we actively investigate, these unspecific predictions are hewn, updated, such that we become increasingly precise at predicting the limits of our bodies. It is thus that the novice becomes the expert, for better or indeed for worse.

In order to better understand the relationship between the inferred state of the body and action, the present enquiry considered the determinants of performance in a sprint task, specifically investigating how the interpretation of altered bodily signals (e.g. heart rate) might influence performance (Watts/Kg). We hypothesised that higher Interoceptive Accuracy in individuals with a high sensitivity to anxiety-related sensations would demonstrate diminished power output.

## Methods

### Participants

Thirty-eight participants (19F; Mean age: 23y; SD±4y) were recruited from a university campus through poster and electronic advertisement, which explicitly noted the requirement for participants to be inactive (description below). All participants were screened for their health and activity level via the International Physical Activity Questionnaire (IPAQ short form) prior to the initiation of the study: only participants determined to be inactive (performed less than 3 x 20 minutes of vigorous exercise per week; <600 MET minutes) and otherwise healthy met the inclusion criteria (Total screened = 40; two excluded due to activity levels). Participants were excluded if they had previously completed the maximal exercise tasks, were currently experiencing pain or had a history of pain lasting more than 3 months (full exclusion criteria: diagnosed cardiovascular disease, diagnosed respiratory disorder (COPD; cystic fibrosis) or neurological disorder (TIA, infarction/haemorrhage, cerebral palsy or neurological deficit), diagnosed psychiatric disorders (specifically, major depressive disorder; schizophrenia or bipolar; other anxiety disorders were not constituted as part of the exclusion criteria). This study was conducted in accordance with the declaration of Helsinki and ethical approval was obtained from divisions of Health (Research Ethics Approval Committee Health, University of Bath) and Psychology (Psychology Ethics Committee, University of Bath). All participants provided written, informed consent. All 38 participants provided written, informed consent.

### Procedure

Participants were required to complete two separate sessions: first a baseline session and then a test session, with a minimum of 24 hours’ rest between each one. For the total duration of both sessions, participants wore a heart rate monitor, which was connected to a Polar V800 watch. All participants were asked to abstain from alcohol and caffeine in the preceding 24-hours of the baseline and test sessions.

#### Baseline session

The purpose of the baseline session was twofold: to establish an objective measure of cardiorespiratory fitness for each participant for the purposes of descriptive analysis and to expose each participant to the same maximal exertion ahead of the test session. The baseline session therefore consisted of a maximal incremental exercise test to volitional exhaustion (VO_2_max test). The VO_2_max test took place in a human physiology laboratory using a static exercise bike (Excalibur Sport, Lode, Groningen, the Netherlands) and an online gas analyser (Parvo TrueOne 2400, Sandy, UT, US). The maximal exercise procedure involved a 2-minute warm up at 50 W, followed by an incremental increase in resistance of 1 W every 3 seconds. Values for VO_2_max were accepted if two or more of the following criteria were met: (1) volitional exhaustion, (2) Respiratory Exchange Ratio (RER)> 1.15, and (3) maximal heart rate within 10 beats of the age-[=predicted maximum (i.e. 220-age)].

#### Test session

The test session comprised four elements: (i) two questionnaires, (ii) a heartbeat counting task, (iii) a 30 second Wingate sprint on a static bike, and (iv) a semi-structured interview.

#### Self-report measures

Two self-report questionnaires were completed in the test session, all components of the Anxiety Sensitivity Index-3 (ASI-3) [34], including physical, social and cognitive concerns. Three components of the Body Perception Questionnaire [35]: Awareness (1), Stress Response (2) and Autonomic Nervous System Reactivity all of which were considered relevant to the present enquiry.

#### Interoceptive accuracy

There exists much controversy in establishing a valid measure of interoceptive accuracy [14]. The present study chose to adopt the most pervasive measure in the literature, the heart beat counting task [36]. Despite identified limitations [37–39], this method allowed us to pursue our primary aim: to call into question the implications that have been drawn from previous studies, specifically that Interoceptive Accuracy, in and of itself, directly influences action.

Participants undertook the heartbeat counting task, which involved 3 heartbeat counting phases, the durations of which were randomised and unknown to the participant (25, 35 and 45 seconds). This test was performed in accordance with the Mental Tracking Method suggested by Schandry [36], in which participants were instructed to focus their attention on their body and count only the heart beats that they were aware of. They performed this task silently and were then asked to verbally report the number of heartbeats at the end of each timed period. The beginning and end of the counting intervals were denoted by an acoustic signal. Interoceptive accuracy was calculated for each time interval, according to the transformation below. A mean score was derived for each participant from the sum across all 3 time intervals; the closer the score to 1, the higher the accuracy.

$$1-\sum \left(\frac{Actual\;HR-Perceived\;HR}{Actual\;HR}\right)$$

Throughout the task participants wore a heart rate monitor attached to a Polar V800 watch, from which the number of actual heartbeats in each interval was derived. The participants were unable to view their heart rate at any time. Using the PolarFlow platform, heart rate data was presented visually and raw data was exported, including the number of actual heart beats within each time interval [40]. Each interval was timestamped in real time, corresponding to the acoustic signal that was provided at the beginning and end of each counting period.

#### Wingate sprint

The Wingate sprint took place in a multipurpose physiology laboratory. Participants were instructed to cycle “at their maximum for 30 seconds”, without pacing (Monark 839E, Varborg, Sweden). Prior to the Wingate sprint, each participant completed a 1-minute warm up at 60 W. The experimenter counted down the final 10 seconds of the warm up and all participants were instructed to start pedalling as fast as they could when the experimenter reached “one”. At zero, the commencement of the Wingate sprint, a resistance equivalent to 7.5% of body mass was applied. Power output was recorded throughout the sprint. After completing the 30-second sprint, participants performed a one-minute cool down at 60 W. The session was complete when the participant reported that they had returned to the condition they were in prior to performing the sprint.

#### Qualitative enquiry

Following the sprint exercise bout, all participants were asked the following questions: 1. “Do you feel that you performed at your maximum in the exercise bout?” 2. “What aspects of your body were you most aware of throughout the exercise bout?” 3. “What do you feel was the limiting factor to your performance in the exercise bout? The inclusion of these qualitative enquiries was to further ascertain the experience of the individual when undergoing an exercise assault, providing context to the current investigation and informing future investigation.

## Statistical analysis

The data from 38 participants were analysed using PASW Statistics (v24.0.0; IBM Corporation, NY). Firstly, bivariate correlation analyses were performed to determine any confounding effects (S1 Tables). Then a moderation analysis was specifically conducted, with Interoceptive Accuracy (predictor variable), Power Output (dependent variable) and Anxiety Sensitivity (moderator variable), using model 1 of the PROCESS add-on tool for SPSS [41].

### Results

#### Preliminary analyses

Table 1 shows the mean and spread of the data for the measures of interoceptive accuracy, anxiety sensitivity and physical performance. Data were normally distributed, and in line with previous studies [9,12].

**Table 1:** Descriptive statistics. Grey shading denotes measures taken from the baseline session, all others were acquired during the test session.

BMI	22.07±2.43 (17.5–28.3)
VO_2_max (mL·kg^-1^·min^-1^)	35±9 (20–55)
Interoceptive Accuracy	52±26 (3–98)
Body Perception Questionnaire	29±14 (8–61)
Anxiety Sensitivity Index-3	23±8 (5–39)
Resting heart rate (beats·min^-1^)	79±10 (58–107)

Values shown are mean ±SD (range)

Next, we explored to what extent Interoceptive Accuracy related to potential confound variables. We determined that Interoceptive Accuracy was not significantly correlated with age, resting heart rate or VO_2_max. In addition, we looked at order and duration effects of the counting intervals, neither of which demonstrated significant difference in interoceptive accuracy scores. We also considered the relationship between the quantitative measures of interoception, one that related to accuracy (Interoceptive Accuracy) and the other that was specific to sensibility (Porges Body Perception Questionnaire [42]; we did not find a significant relationship between these variables. We did, however, find a significant correlation between the ASI-3 and the Porges Body Perception Questionnaire, suggestive of the key role for the interpretation of bodily events (S1 Tables for further analysis of key variables, including sub-score analysis, which was not powered for in the main study).

### Moderation analysis

**Fig 2:**
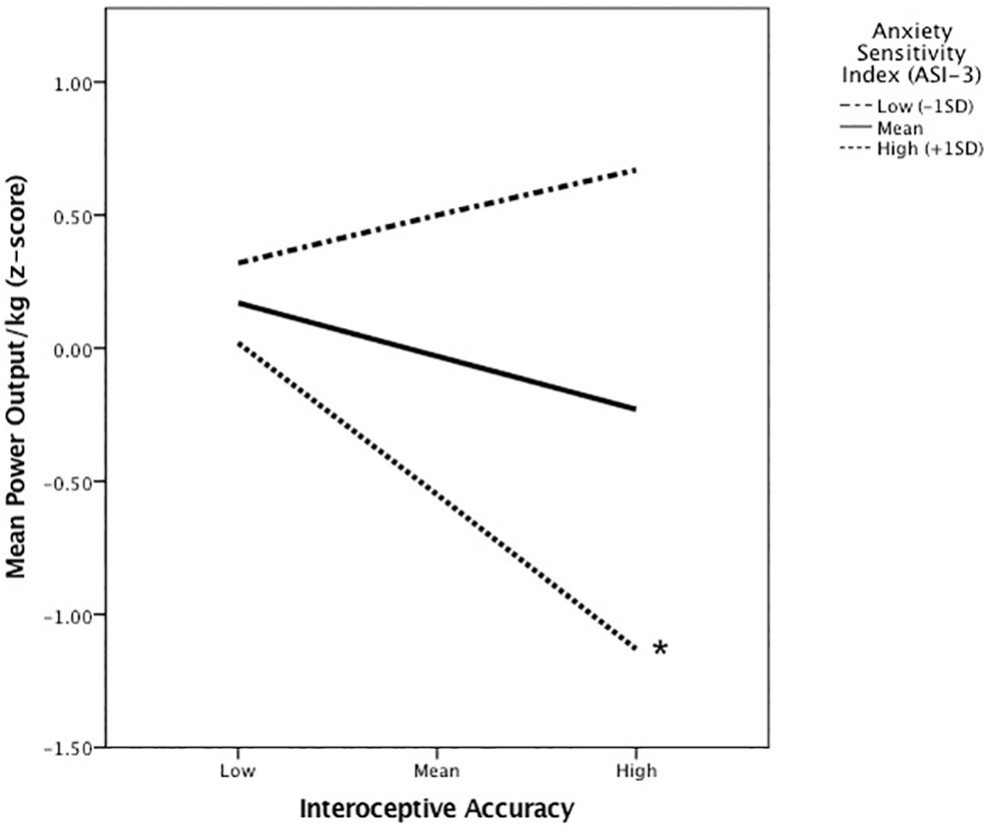
Moderation analysis: Interoceptive accuracy, anxiety sensitivity and mean power output. *Denotes significant interaction.

For the moderation analysis, Interoceptive Accuracy was the predictor variable, mean power output (corrected for body mass: Watts/Kg) was the outcome variable and Anxiety Sensitivity index was the moderator variable. The overall moderation model was significant (*R*^*2*^ = 0.47; *F*(3, 34) = 17.37; *p*<0.001). Interoceptive Accuracy was not a significant predictor of mean power output (*p = 0*.*07*). Anxiety Sensitivity was a significant predictor of mean power output (*p<0*.*001)*. Importantly, the interaction between Interoceptive Accuracy and Anxiety Sensitivity was significant, indicating that there was a moderation effect. Specifically, the moderation effect by Anxiety Sensitivity on the relationship between Interoceptive Accuracy and performance accounted for a statistically significant difference in mean power output (Δ*R*^*2*^ = 0.19; *F*(1,34) = 19.87); *p*<0.001) (see Fig 2); At low levels of AS (-1 SD) there was no significant relationship between Interoceptive Accuracy and mean power output (*b* = 0.88, 95% CI [-0.77, 2.52], *t* = 1.08, *p* = 0.29). At mean levels of Anxiety Sensitivity there was no significant relationship between Interoceptive Accuracy and mean power output (*b* = -0.99; 95% CI [-2.07, 0.08], *t* = -1.87, *p* 0.07). However, at high levels of Anxiety Sensitivity (+ 1SD) there was a significant negative relationship between Interoceptive Accuracy and mean power output (*b* = -2.87, 95% CI [-0.33, -0.12], *t* = -4.46, *p* = 0.001).

### Exploratory analysis: Effect of sex of participant

**Fig 3:**
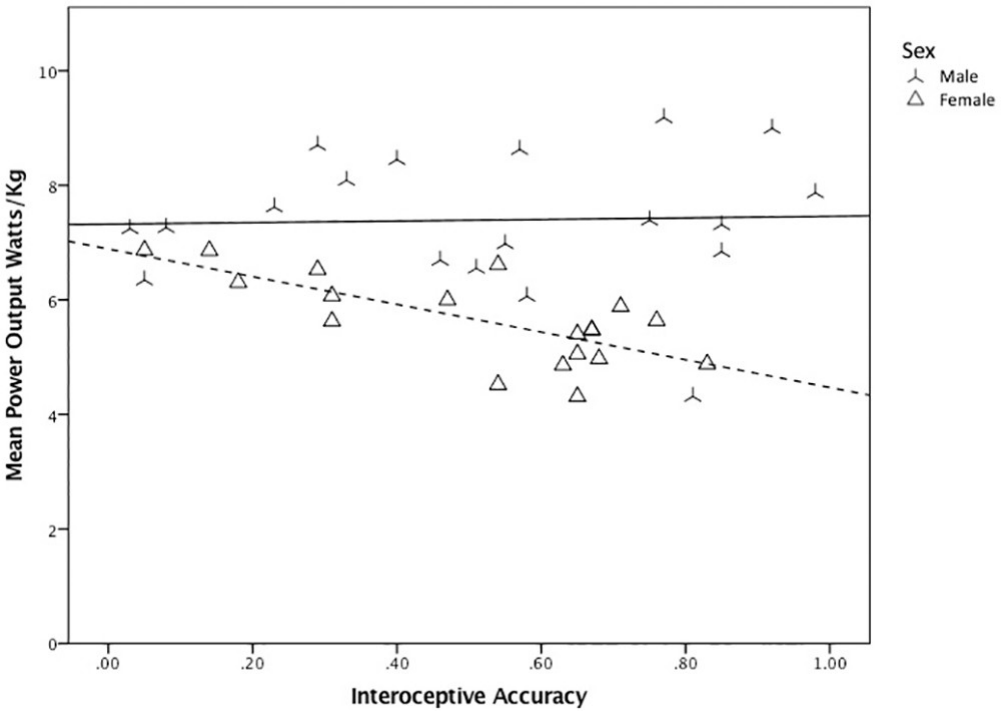
The relationship between interoceptive accuracy and mean power output across all participants, separated by sex.

Interoceptive Accuracy, across all participants, was not significantly correlated with mean Power Output Watts/Kg (r(36) = -0.141, *p =* 0.398. However, in women, Interoceptive Accuracy was significantly correlated with mean Power Output (*R*^*2*^ = 0.52, *p*<0.001; whereas no such relationship was observed in men (*R*^*2*^ = 0.001, *p* = 0.888) (Fig 3). Relevant to the main result of this investigation, the subset of participants within the high anxiety sensitivity group was comprised of 11 females and 7 males. For further investigation into sex differences, refer to S1 Tables.

### Qualitative enquiry

All participants confirmed that they judged their performance to be at maximum during the exercise challenge. In response to question 2, the majority (n = 29) commented that they were most aware of a ‘heaviness’, ‘fatigue’ or ‘dull ache’ in their legs. A small proportion noted that they were aware of their heart rate (n = 4) and breathing rate (n = 6) during the task. Eighteen of the participants reported that it was only after they had stopped exercising that they had become aware of their heart rate and breathing rate. For the majority of participants, the limiting factor to performance was the various sensations associated with the legs. Two individuals noted that temperature was the bodily sensation they were most aware of.

## Discussion

The aim of this study was to examine whether Interoceptive Accuracy and Anxiety Sensitivity interact to predict performance in a sprint exercise task. Our primary finding supports our hypothesis, which specified that performance in a sprint-based exercise task is determined by both the ability to accurately detect bodily changes as well as the level of anxiety sensitivity in association with such changes. These results confirm that in a group of inactive individuals, Interoceptive Accuracy alone does not predict the actions taken when participants are exposed to an unfamiliar exercise task. In fact, the impact of Interoceptive Accuracy on performance was moderated by the level of Anxiety Sensitivity expressed by the participant. Specifically, at high levels of Anxiety Sensitivity there was a significant negative relationship between Interoceptive Accuracy and performance. In other words, we found that individuals who demonstrated elevated accuracy in detecting arousal-related changes, specifically related to heart rate, *and* who showed a heightened fear of the consequences of these arousal symptoms, altered their actions accordingly: decreasing their mean power output in a sprint task.

Previous investigation has noted the importance of taking into account the accuracy by which we detect our internal bodily condition; this work has highlighted Interoceptive Accuracy as a having a direct influence on protection, enhancing behavioural regulation, whether in exercise [9], or in relation to pain [8]. Relevant to this, Pollatos et al, (2007), identified that individuals with high cardiovascular reactivity, showed increased trait anxiety and interoceptive accuracy [43]. Our present findings offer an insight into the nature of this relationship and how it potentially curbs future action. This work draws together evidence across the fields of psychology and embodied cognition, providing additional support for the position taken by Smits et al., who have highlighted not only the role of anxiety sensitivity in relation to physical activity adherence [44,45] but also the protective role of physical activity in the development of anxiety disorders [46].

Our results were unable to proffer support for a direct relationship between Interoceptive Accuracy and behavioural regulation at a group level. Instead, we found that the inference drawn from arousal-based bodily signals, surmised through Anxiety Sensitivity, was a necessary moderator of performance. From the theoretical position, which drove this investigation, these findings provide a coherent picture: we did not anticipate that accuracy in detecting bodily signals in isolation would alter performance, but rather that performance would be guided by what was inferred from this information. One potential explanation for these discrepancies in findings may be related to the cohorts that were recruited. We deliberately recruited a homogenous group of individuals in relation to their activity levels. In so doing, we exposed them to an unfamiliar sprint exercise task in an attempt to expose unrefined predictions associated with the engagement of the task and the consequences of their actions within it. This consideration has not played a part in previous studies.

Our principal finding has several interesting implications. Broadly, it provides a base from which to better understand the potential mechanisms underlying approach and avoidance behaviours. In the present case, this is particularly relevant to inactive individuals and the effective prescription of exercise and the promotion of healthy living. With the increasing prevalence of high intensity, interval training (HIIT) paradigms, which prioritise the minimisation of barriers to exercise, understanding why high dropout rates still occur is important. We found that in this group of inactive individuals, the interpretation of acute bodily arousal change played a crucial role in the way they performed. Thus, simply prescribing exercise for people who do not currently exercise, may, for some, engage defensive action associated with fears of the consequences of exercise-related arousal. Being able to identify those individuals and thus prescribing exercise in line with individual bodily inferences may help in exercise uptake as well as the maintenance of a long-term active lifestyle. Not accounting for these individual differences in inactive people could contribute to high drop-out rates associated with the fear of the predicted consequences of exercise [47–49]. Extending the findings of this investigation into the interpretation of body-relevant information in other groups of individuals, such as elite athletes and those with injuries (Fig 1b & 1c) will provide further insight into what determines pushing the boundaries of human performance as well as what promotes successful rehabilitation.

Our exploratory analysis exposed an important consideration of sex differences. This sits in line with previous literature, which asserts the multifactorial nature of interoception [50], accounting for the impact of sex differences is integral to furthering our understanding. Although the study was not powered to detect sex of participant effects, our results imply that further investigation is justified. From our exploratory findings, it is suggested that interoceptive accuracy may indeed have a direct influence on performance in women but not in men. Given that there was a predominance of women within the high Anxiety Sensitivity group, and considering increased prevalence of anxiety disorders in women as compared to men, deciphering the influences on physical activity between sexes becomes a target for future research. This could prove a fruitful line of enquiry when considering the impact of accurately inferring bodily state not only in physical activity but also in conditions that involve chronic protective behaviours, such as pain, fatigue, panic and nausea [26].

We conducted our study in inactive participants, naïve to the process of exercise testing. This was a deliberate attempt to place individuals in a context that was unfamiliar to them and therefore one that they had a limited array of predictions regarding the consequences of their actions. Our analysis reflected this priority in considering the impact of high and low Interoceptive Accuracy and Anxiety Sensitivity on performance, centred around the mean. However, it will be important to extend this investigation to other groups of people. Considering how individuals act in their environment when faced with bodily perturbation is relevant to a host of bodily experiences, from fatigue to pain, but including thermo-perception, and under-explored bodily sensations such as tumescence, stiffness, and perception of limb weight [28]. The extension of this work is required to add rigor to our finding, not only within other populations but across a greater number of individuals, as our study, despite being powered to detect the anticipated effect, is limited in its translation due to the modest sample size.

The quantification of interoceptive accuracy remains a challenge to the field, and one that has not specifically been addressed here. The heart beat counting task has been widely used but the validity of the measure has called into question in light of potential confounding factors: attention, beliefs, expectations [37,38,51,52]. There is a necessity to replicate the present findings in relation to other objective measures of interoceptive accuracy both within and outside of the cardiovascular domain.

## Conclusion

Our findings highlight that the predicted consequences of action in line with an ability to accurately identify bodily changes should be accounted for when considering the determination of bodily limits. These results provide a first investigation of how limits of performance are negotiated in uncertain situations.

## Supporting information

S1 Tables Exploratory analyses. (DOCX)
