# Supplementary material for: Predicting the consequences of physical activity: An investigation into the relationship between anxiety sensitivity, interoceptive accuracy and action
Source: PLoS One. 2019 Mar 28;14(3):e0210853. doi: 10.1371/journal.pone.0210853 (PMC6438567; doi:10.1371/journal.pone.0210853)
Supplement: S1 Tables — (DOCX) [file pone.0210853.s001.docx]

Exploratory Analyses

**Bivariate Correlation Analyses**

|  | | | | | | | |
| --- | --- | --- | --- | --- | --- | --- | --- |
|  | | MeanPO/Kg | ASI-3 | IA | RER Max | PBQ | HR  Variability |
| MeanPO/Kg | Pearson Correlation | 1 | **-.501^**^** | -.141 | .072 | -.217 | -.162 |
|  | Sig. (2-tailed) |  | .001 | .398 | .669 | .191 | .332 |
|  |  |  |  |  |  |  |  |
| ASI-3 | Pearson Correlation | **-.501^**^** | 1 | -.073 | -.273 | .**547^**^** | .022 |
|  | Sig. (2-tailed) | .001 |  | .663 | .097 | .000 | .894 |
|  |  |  |  |  |  |  |  |
| IA | Pearson Correlation | -.141 | -.073 | 1 | .111 | .010 | .175 |
|  | Sig. (2-tailed) | .398 | .663 |  | .507 | .954 | .295 |
|  |  |  |  |  |  |  |  |
| RER  Max | Pearson Correlation | .072 | -.273 | .111 | 1 | -.006 | -.137 |
|  | Sig. (2-tailed) | .669 | .097 | .507 |  | .972 | .413 |
|  |  |  |  |  |  |  |  |
| PBQ | Pearson Correlation | -.217 | **.547^**^** | .010 | -.006 | 1 | .116 |
|  | Sig. (2-tailed) | .191 | .000 | .954 | .972 |  | .487 |
|  |  |  |  |  |  |  |  |
| HR  Variability | Pearson Correlation | -.162 | .022 | .175 | -.137 | .116 | 1 |
|  | Sig. (2-tailed) | .332 | .894 | .295 | .413 | .487 |  |
|  |  |  |  |  |  |  |  |
| **. Correlation is significant at the 0.01 level (2-tailed). | | | | | | | |

Table Glossary

MeanPO/KG (Mean Power Output / Kg)

ASI-3 (Anxiety Sensitivity Index-3)

IA (Interoceptive Accuracy)

RER Max (Respiratory Exchange Ratio Max)

PBQ (Porges Body Perception Questionnaire)

HR Variability (root mean squared of successive difference of intervals (RMSSD))

**Sub-score Analyses (ASI-3 and Porges Body Perception Questionnaire)**

|  | | | | | | | |
| --- | --- | --- | --- | --- | --- | --- | --- |
|  | | ASIphy | ASIcog | ASIsoc | Porges  1 | Porges  2 | Porges  3 |
| ASIphy | Pearson Correlation | 1 | .187 | -.111 | .286 | .201 | .180 |
|  | Sig. (2-tailed) |  | .260 | .509 | .081 | .226 | .281 |
|  |  |  |  |  |  |  |  |
| ASIcog | Pearson Correlation | .187 | 1 | .281 | **.592^**^** | **.568^**^** | **.616^**^** |
|  | Sig. (2-tailed) | .260 |  | .087 | .000 | .000 | .000 |
|  |  |  |  |  |  |  |  |
| ASIsoc | Pearson Correlation | -.111 | .281 | 1 | .085 | .235 | .233 |
|  | Sig. (2-tailed) | .509 | .087 |  | .613 | .156 | .159 |
|  |  |  |  |  |  |  |  |
| Porges  1 | Pearson Correlation | .286 | .**592^**^** | .085 | 1 | .**655^**^** | .**773^**^** |
|  | Sig. (2-tailed) | .081 | .000 | .613 |  | .000 | .000 |
|  |  |  |  |  |  |  |  |
| Porges  2 | Pearson Correlation | .201 | **.568^**^** | .235 | **.655^**^** | 1 | **.696^**^** |
|  | Sig. (2-tailed) | .226 | .000 | .156 | .000 |  | .000 |
|  |  |  |  |  |  |  |  |
| Porges  3 | Pearson Correlation | .180 | **.616^**^** | .233 | .**773^**^** | **.696^**^** | 1 |
|  | Sig. (2-tailed) | .281 | .000 | .159 | .000 | .000 |  |
|  |  |  |  |  |  |  |  |
| **. Correlation is significant at the 0.01 level (2-tailed). | | | | | | | |

**Alternative Moderation Analysis**

1. **Moderator: ASI-3 Physical concerns**

|  | Coeff | se | t | p | LLCI | ULCI |
| --- | --- | --- | --- | --- | --- | --- |
| Constant | 6.5788 | 0.1899 | 34.6394 | 0.000 | 6.1783 | 6.9793 |
| IA | -0.5734 | 0.6878 | -0.8338 | 0.4102 | -1.9712 | 0.8243 |
| ASI-3 Physical | -0.1875 | 0.0717 | -2.6157 | 0.0132 | -0.3332 | -0.0418 |
| Int_1 | -0.4422 | 0.2174 | -2.5237 | **0.0498** | -0.8841 | -0.0003 |

1. **Moderator: ASI-3 Cognitive concerns**

|  | Coeff | se | t | p | LLCI | ULCI |
| --- | --- | --- | --- | --- | --- | --- |
| Constant | 6.5675 | 0.1792 | 36.6557 | 0.000 | 6.2033 | 6.9316 |
| IA | -0.4718 | 0.6201 | -0.7608 | 0.4520 | -1.7319 | 0.7884 |
| ASI-3 Cognitive | -0.1598 | 0.0413 | -3.8724 | 0.0005 | -0.2437 | -0.0759 |
| Int_1 | -0.4167 | 0.1139 | -3.6576 | **0.0009** | -0.6482 | -0.1852 |

1. **Moderator: ASI-3 Social concerns**

|  | Coeff | se | t | p | LLCI | ULCI |
| --- | --- | --- | --- | --- | --- | --- |
| Constant | 6.4475 | 0.2242 | 28.7536 | 0.000 | 5.9918 | 6.9032 |
| IA | -1.1426 | 0.7772 | -1.4701 | 0.1507 | -2.7222 | 0.4369 |
| ASI-3 Social | -0.0729 | 0.0412 | -1.7687 | 0.0859 | -0.1567 | -0.0109 |
| Int_1 | -0.4167 | 0.1139 | -3.6576 | 0.1073 | -0.4619 | -0.1852 |

**Sex Differences**

| **Independent Samples Test** | | | | | | | | | |
| --- | --- | --- | --- | --- | --- | --- | --- | --- | --- |
|  | | t | df | Sig.  (2-tailed) | Mean Difference | Std. Error Difference | 95% Confidence Interval of the Difference | |  |
|  |  |  |  |  |  |  | Lower | Upper |  |
| ASI-3 |  | -1.254 | 36 | .218 | -3.36842 | 2.68610 | -8.81609 | 2.07925 |  |
| IA |  | .000 | 36 | 1.000 | .00000 | .08908 | -.18067 | .18067 |  |
| MeanPOKg |  | 5.405 | 36 | **.000** | 1.74417 | .32267 | 1.08976 | 2.39858 |  |
| VO2MaxRE |  | 5.460 | 36 | **.000** | 11.41263 | 2.09023 | 7.17345 | 15.65181 |  |
